# 78 Improvement of Cell Viability in Temporary Storage of Split Thickness Skin Grafts

**DOI:** 10.1093/jbcr/irad045.052

**Published:** 2023-05-15

**Authors:** Brandon Couch, Maria Batchinsky, Alan Pang, Irfan Warraich, Michael Melkus, Seungman Kim, Deepak Bharadia, John Griswold

**Affiliations:** Texas Tech University Health Sciences Center School of Medicine, Lubbock, Texas; Texas Tech University Health Sciences Center School of Medicine, Lubbock, Texas; Texas Tech University Health Sciences Center, Lubbock, Texas; Texas Tech University Health Sciences Center, Lubbock, Texas; Texas Tech University Health Sciences Center, Lubbock, Texas; Texas Tech University Health Sciences Center, Lubbock, Texas; Texas Tech University Health Sciences Center, Lubbock, Texas; Texas Tech University Health Sciences Center, Lubbock, Texas

## Abstract

**Introduction:**

Autologous split thickness skin grafts (STSGs) are the preferred donor tissue for restoration of large tissue deficits. Although grafting should be done at the time of donor harvest, various factors necessitate tissue storage, traditionally in saline solution (SS) at 4^o^C. However, STSGs often expire before grafting in these conditions. Patients then require further excision procedures or die due to inadequate skin coverage. For patients’ wellbeing, establishing a new storage protocol is imperative. This study sought to determine an effective storage media for prolongation of graft viability for subsequent incorporation into excision and grafting practice.

**Methods:**

Following patient consent, excess tissue >1cm^2^ was collected after grafting procedures. Tissue was stored at 4^o^C in RPMI, DMEM, and SS with penicillin/streptomycin. Samples were evaluated at 2, 5, 7, and 14 days. Cell viability was measured by Trypan blue dye exclusion test in an automated cell counter yielding percentage of viable keratinocytes (PVK). An unbiased pathologist assessed tissue integrity by epidermal-dermal junction, perinuclear keratinocyte halos, and collagen organization with higher scores denoting deterioration. Data were evaluated by ANOVA and Effect size.

**Results:**

A total of 155 sets of two specimens each were collected from 13 patients. Mean PVK values at day 2, 5, 7, and 14: SS (57.38, 36.96, 38.38, 27.50), RPMI (69.46, 69.92, 70.33, 62.67), DMEM (63.17, 53.50, 54.83, 37.92). By pairwise comparison, RPMI preserved PVK better than SS at days 5, 7, and 14 (p = 0.001 for each day). DMEM preserved PVK better than SS when comparing all days (p = 0.011) but failed to produce a statistically significant difference at any specific day. RPMI preserved PVK greater than DMEM each day. However, statistically significant differences were only revealed at day 14 (p = 0.017) and when comparing all days (p < 0.001). Effect size was large for overall group differences of PVK at day 5, 7, and 14 (Cohen’s f = 0.72, 0.73, and 0.75). Tissue integrity scores at day 2, 5, 7, and 14: SS (0.69, 1.0, 1.38, 2.0), RPMI (0.54, 0.92, 0.85, 1.54), DMEM (0.77, 0.92, 0.77, 1.15). Although culture media preserved tissue integrity greater than SS, no statistically significant differences were found.

**Conclusions:**

RPMI preserved cell viability better than DMEM and SS. This suggests that STSGs stored in RPMI may prolong the time of which tissue could be stored and successfully amalgamated with skin.

**Applicability of Research to Practice:**

Storing tissue in RPMI could benefit burn patients by decreasing tissue disposal therefore reducing future donor site area or eliminating repetitious excision procedures entirely. These effects decrease inpatient days and healthcare costs. Most importantly, successful tissue storage will save patients’ lives, especially those of large surface area burns who lack sufficient donor sites.

