# Correction to: 78 Improvement of Cell Viability in Temporary Storage of Split Thickness Skin Grafts

**DOI:** 10.1093/jbcr/irad147

**Published:** 2023-10-04

This is a correction to: Brandon Couch, Maria Batchinsky, Alan Pang, Irfan Warraich, Michael Melkus, Seungman Kim, Deepak Bharadia, John Griswold, 78 Improvement of Cell Viability in Temporary Storage of Split Thickness Skin Grafts, Journal of Burn Care & Research, Volume 44, Issue Supplement_2, May/June 2023, Pages S42–S43, https://doi.org/10.1093/jbcr/irad045.052

In the originally published version of this abstract, there was a typographical error in the name of author Maria Batchinsky.

This has been corrected.

